# Reliability of the Multidimensional Pain Inventory and stability of the MPI classification system in chronic back pain

**DOI:** 10.1186/1471-2474-13-155

**Published:** 2012-08-24

**Authors:** Martin L Verra, Felix Angst, J Bart Staal, Roberto Brioschi, Susanne Lehmann, André Aeschlimann, Rob A de Bie

**Affiliations:** 1 Department of Physiotherapy, Inselspital, Bern University Hospital, 3010, Bern, Switzerland; 2 Rehabilitation clinic “RehaClinic”, 5330, Bad Zurzach, Switzerland; 3 Department of Epidemiology and Caphri Research School, Maastricht University, Maastricht, The Netherlands; 4 Scientific Institute for Quality of Healthcare, Radboud University Medical Centre, Nijmegen, The Netherlands

**Keywords:** Reliability, Back pain, Cluster, Subgroup, MPI, Classification

## Abstract

**Background:**

This cross validation study examined the reliability of the Multidimensional Pain Inventory (MPI) and the stability of the Multidimensional Pain Inventory Classification System of the empirically derived subgroup classification obtained by cluster analysis in chronic musculoskeletal pain. Reliability of the German Multidimensional Pain Inventory was only examined once in the past in a small sample. Previous international studies mainly involving fibromyalgia patients showed that retest resulted in 33–38% of patients being assigned to a different Multidimensional Pain Inventory subgroup classification.

**Methods:**

Participants were 204 persons with chronic musculoskeletal pain (82% chronic non-specific back pain). Subgroup classification was conducted by cluster analysis at 4 weeks before entry (=test) and at entry into the pain management program (=retest) using Multidimensional Pain Inventory scale scores. No therapeutic interventions in this period were conducted. Reliability was quantified by intraclass correlation coefficients (ICC) and stability by kappa coefficients (κ).

**Results:**

Reliability of the Multidimensional Pain Inventory scales was least with ICC = 0.57 for the scale life control and further ranged from ICC = 0.72 (negative mood) to 0.87 (solicitous responses) in the other scales. At retest, 82% of the patients in the Multidimensional Pain Inventory cluster interpersonally distressed (κ = 0.69), 80% of the adaptive copers (κ = 0.58), and 75% of the dysfunctional patients (κ = 0.70) did not change classification. In total, 22% of the patients changed Multidimensional Pain Inventory cluster group, mainly into the adaptive copers subgroup.

**Conclusion:**

Test-retest reliability of the German Multidimensional Pain Inventory was moderate to good and comparable to other language versions. Multidimensional Pain Inventory subgroup classification is substantially stable in chronic back pain patients when compared to other diagnostic groups and other examiner-based subgroup Classification Systems. The MPI Classification System can be recommended for reliable and stable specification of subgroups in observational and interventional studies in patients with chronic musculoskeletal pain.

## Background

The search for homogeneous subgroups of patients with nonspecific low back pain who respond best to subgroup-specific pain management interventions has been on the international research agenda for over 15 years [1]. The process of developing treatment-based subgroups can be divided into 3 stages: 1) hypothesis generation; proposal of potential effect modifiers; 2) hypothesis testing; testing of the potential effect modifiers; 3) replication; assessing generalizability [2]. Several physiotherapy-based classification systems for low back pain have been developed [3,4]. So far, most subgrouping approaches have been based on unproven theories, are poorly validated or remain, as yet, unreplicated in other studies [5]. Besides biological factors of low back pain and spinal movement or mechanical loading strategies, especially in chronic pain syndromes psychosocial factors are also likely to modify treatment response. As such, investigation of effect modifiers from the full biopsychosocial spectrum seems the most likely way to identify clinically important subgroups [6].

The Multidimensional Pain Inventory is a self-report instrument assessing not only pain intensity and pain interference, but also the way people cope with pain, it measures support as well as potential reinforcement of pain behaviors by the patient’s significant other, and peoples’ general activity level. The Multidimensional Pain Inventory has been translated into several languages and validated in various settings for several diagnostic pain groups [7,8]. The reliability of the German version of the Multidimensional Pain Inventory has been tested in a relatively small sample by only one research group for over 20 years [9]. An approach based on cluster analysis of the mean scores of the scales of the Multidimensional Pain Inventory yielded three unique profiles or subgroups for patients with chronic pain [10]. So far, two research groups assessed test-retest stability of the MPI Classification System [11-13]. In their samples of patients with low back pain and fibromyalgia up to one third of the patients changed Multidimensional Pain Inventory classification at retest. The authors concluded that for a sizeable number of chronic pain patients, Multidimensional Pain Inventory subgroup classifications may not be stable and need to be reconsidered [11,13].

The main aim of the present study was to re-examine the test-retest stability of the MPI Taxonomy Classification in patients with persistent musculoskeletal pain. The secondary objective focussed on the elaboration of additional evidence concerning internal consistency of items within scales and test-retest reliability at scale level of the German version of the Multidimensional Pain Inventory.

## Methods

### Setting and participants

The study was conducted at the rehabilitation clinic “RehaClinic” (locations Bad Zurzach and Braunwald, Switzerland), which is attended by severely disabled patients suffering from persistent musculoskeletal pain. The patients were assessed prior to participating in the “Zurzach Interdisciplinary Pain Program” - ZISP. The program is a 4-week in-house, standardized, interdisciplinary pain management program. All subjects were consecutively admitted and included in the study and 1) suffered either from chronic non-specific back pain (i.e. lumbar, thoracic, or pan-vertebral pain syndrome without serious spinal pathology or nerve root pain) or suffered from fibromyalgia according to the original American College of Rheumatology criteria, 2) had pain for at least 6 months and 3) were disabled by their pain enough to warrant admission to an intensive inpatient pain management program [14,15]. Further inclusion criteria were 4) ability to complete self-assessment questionnaires, 5) understand the German language, 6) no psycho-intellectual inabilities, and 7) provision of written, signed informed consent. Exclusion criteria were 1) severe somatic illness requiring specific treatment such as cancer, inflammatory rheumatic disease, neurological disease, and pain after a recent operation, 2) manifest psychiatric disorder such as dementia, psychosis, suicidality, and 3) failed inclusion criteria.

The study design is a cohort study with 4 weeks follow-up. The study protocol was approved by the Local Ethic Commission (Health Department in Aarau, Switzerland, no. EK AG 2008/026). All participants gave written informed consent according to the Declaration of Helsinki.

### Outcome measures

The West Haven-Yale Multidimensional Pain Inventory (MPI) measures multiple aspects of the individual pain experience and comprises three sections with a total of 13 factors analytically derived from scales based on items ranging from 0 to 6 (seven points) [16]. The factor structure has been replicated in several international samples. Kerns and colleagues reported excellent validity, internal consistency, and reliability of this instrument [16]. Results of a study by Junghaenel and Broderick revealed that Multidimensional Pain Inventory ratings obtained from the partner or health care provider corresponded with the self-report patient profiles [12]. The Initiative on Methods, Measurement, and Pain Assessment in Clinical Trials (IMMPACT) recommended the Multidimensional Pain Inventory as a valuable component of a comprehensive assessment tool [17]. The German version of the Multidimensional Pain Inventory is a self-report 51-item inventory with the same eleven scales as the original US version: pain severity, interference due to pain, life control, affective distress (synonymously described as negative mood), support, punishing responses, solicitous responses, distracting responses, social and recreational activities, household chores, and activities away from home [18]. The last three subscales can be summarized into one subscale of general activities. Cronbach’s alpha reliability coefficients vary between α = 0.63–0.93, and test-retest reliability scores ranged from r_p_ = 0.46–0.93 [9]. Comparing 5 assessment instruments for chronic pain, the Multidimensional Pain Inventory was most responsive in all comparable domains [19].

An approach based on cluster analysis of the mean scores of the scales of the Multidimensional Pain Inventory yielded three unique profiles or subgroups for patients with chronic pain. The Multidimensional Pain Inventory differentiates between three subgroups labelled as adaptive copers, dysfunctional, and interpersonally distressed [10]. The interpersonally distressed cluster is mainly characterized by lower levels of perceived solicitous and distraction responses from the patients’ partners or spouses and higher levels of punishing responses compared to the adaptive copers and dysfunctional clusters. The adaptive copers cluster, compared with the other two subgroups, is characterized by less pain severity, less interference with everyday life due to pain and less affective distress, more perception of life control and higher activity level. The persons of the dysfunctional cluster report high pain severity, high interference and activity distress, low life control, and low activity level.

### Statistical analysis

The Multidimensional Pain Inventory was assessed 4 weeks before entry to the clinic and at entry to the clinic (pre-treatment). No therapeutic interventions took place in this period. This time interval is 1) sufficiently short that we can assume that the underlying process of chronic musculoskeletal pain is unlikely to have changed, and 2) sufficiently long that we can assume that the patients did not memorize their item responses of the first occasion.

The internal consistency of the Multidimensional Pain Inventory was assessed by using Cronbach’s alpha, a statistic used to calculate the strength of the association between the individual items within the scale. The alpha coefficient examines inter-item correlations and therefore relates to its homogeneity. Because a Cronbach’s alpha ≥ 0.90 might suggest a high level of item redundancy, ideally Cronbach’s alpha should be above 0.70, but probably not higher than 0.90 [20].

Test-retest reliability of the Multidimensional Pain Inventory scales was determined by intraclass correlation coefficients (ICC). The intraclass correlation coefficient measures the consistency and degree of correspondence of the test and retest responses pairwise for each item and each patient for the whole sample and ranges from 0.00 (no consistency) to 1.00 (perfect consistency). Although the intraclass correlation coefficient is primarily designed for use with interval or ratio data, the intraclass correlation coefficient can be applied without distortion of the data on the ordinal scale of the Multidimensional Pain Inventory when intervals between such measurements are assumed to be equivalent [21]. For comparability with other studies test-retest reliability of the Multidimensional Pain Inventory scales was also determined by Pearson correlation coefficients: 0.00 means no correlation and 1.00 means perfect correlation.

According to Turk and colleagues, the empirically derived subgroups were defined by confirmatory cluster analysis using a predefined three cluster solution [10]. The Multidimensional Pain Inventory score patterns of this study were depicted as graphs of the mean Multidimensional Pain Inventory baseline scale scores and compared to the patterns described by Turk and colleagues and our previous studies on this topic using the rank orders of the three subgroups within one Multidimensional Pain Inventory subscale [22,23].

The test-retest stability of the Multidimensional Pain Inventory clusters was determined by percent of agreement and kappa coefficients. The kappa statistic is a chance-corrected measure of percent agreement for ordinal or nominal scales [24]. It is a useful method for summarizing observer consistency (inter- or intertester reliability) and provides valuable information on the stability of classification procedures used in musculoskeletal practice, for example. The following standards for strength of agreement for the Kappa coefficient have been proposed: <0 = poor, 0.00–0.20 = slight, 0.21–0.40 = fair, 0.41–0.60 = moderate, 0.61–0.80 = substantial and 0.81–1.00 = almost perfect [25].

All analyses were performed using the statistical software package SPSS 20.0 for Windows® (SPSS Inc., Chicago, IL, USA).

## Results

### Participants at baseline

Table 1 describes the demographic and medical data of the total sample of patients with mainly chronic non-specific back pain on entry into the pain management program (n = 204). The subjects were characterized by relatively young age (on average 46.8 years), high level of incapacity for work (50.5%), and a long history of pain (on average, 10.4 years). High scores for pain intensity, interference with pain, and negative mood, and low scores for life control and general activity level complete the profile of these patients in this sample (Table 2).

**Table 1:** Demographic characteristics of the sample (n = 204)

**Dimension**	
Diagnosis chronic back pain (%)	82
Diagnosis fibromyalgia (%)	15
Diagnosis, other (%)	3
Average age, min-max (years)	46.8 (16.7–72.8)
Average beginning of symptoms, min-max (years)	10.4 (0.25–64.5)
Sex: female (%)	71.1
Education: none (%)	1
Education: Grade 10–12 (%)	31
Education: High school graduate (%)	55
Education: College graduate (%)	9
Education: University graduate (%)	4
Employment status: inability for work (%)	50.5
Employment status: full-time (%)	28.9
Smoking: yes (%)	43
Marital status: single (%)	23
Marital status: married (%)	66
Marital status: other (%)	11

**Table 2:** Test-retest reliability of mean MPI scale scores and Internal consistency of the items at scale level for all subjects (n = 204)

**MPI subscales**	**T0 (m,s)**	**T1 (m,s)**	**ICC (95% CI)**	**CA (95% CI)**
Pain severity (100 = most)	76.1 (16.5)	73.9 (16.0)	0.77 (0.70–0.82)	0.81 (0.77–0.86)
Interference (100 = worst)	74.7 (15.9)	72.5 (16.1)	0.82 (0.77–0.86)	0.83 (0.76–0.86)
Life control (100 = best)	49.4 (22.1)	50.2 (21.4)	0.57 (0.47–0.66)	0.76 (0.69–0.81)
Negative mood (100 = worst)	61.0 (19.6)	58.4 (22.1)	0.72 (0.65–0.78)	0.60 (0.49–0.69)
Support (100 = most)	69.4 (24.8)	69.0) 26.2)	0.85 (0.80–0.88)	0.82 (0.77–0.86)
Negative responses (100 = most)	25.6 (26.1)	24.3 (24.4)	0.75 (0.69–0.81)	0.86 (0.82–0.89)
Solicitous responses (100 = best)	58.3 (25.0)	58.6 (25.0)	0.87 (0.83–0.90)	0.81 (0.76–0.85)
Distracting responses (100 = best)	53.6 (25.3)	55.3 (24.8)	0.77 (0.71–0.82)	0.69 (0.61–0.76)
General activity (100 = most)	34.5 (13.7)	35.1 (13.4)	0.86 (0.82–0.90)	0.82 (0.78–0.86)

MPI: Multidimensional Pain Inventory, CA: Cronbach’s alpha, 95% CI: 95% confidence interval, T0: 4 weeks before entry to pain management program, T1: at entry to pain management program, m: mean, s: standard deviation, ICC: intraclass correlation coefficient.

### Internal consistency and test-retest reliability of the MPI scales

Cronbach’s alpha was measured for 7 out of 9 Multidimensional Pain Inventory scales with scores between 0.76 and 0.86 and so reflects a good association between the individual items within their scales. The internal consistency of the items within the scales negative mood and distracting responses was smaller (0.60, and 0.69, resp.). Test-retest reliability, measured at an average 4-week time interval, for the mean Multidimensional Pain Inventory scale scores was very good with scores between ICC = 0.72 and 0.87. Only the score for the MPI scale life control (ICC = 0.57) was less favourable (Table 2).

### Classification and test-retest stability of patients in the Multidimensional Pain Inventory subgroups

All 204 patients could be allocated by cluster analysis into one of the three predefined chronic pain subgroups at both time points. The Multidimensional Pain Inventory scores differed significantly between the three clusters: the patients in the dysfunctional cluster showed highest scores for pain severity, interference due to pain, and negative mood and lowest scores for general activities. In accordance with the predefined profile of the empirically derived MPI Classification System, the cluster interpersonally distressed showed lowest scores for support, solicitous and distracting responses by their partner or spouses, and the highest score for negative/ punishing responses by their partner or spouses. Compared to the other two subgroups, the adaptive copers showed best scores for life control, negative mood, and general activities (Table 3).

**Table 3:** Mean scores and standard deviations of the scales of the Multidimensional Pain Inventory clusters at 4 weeks before and at entry to a pain program

**MPI scales**	**MPI clusters at T0 (m,s)**			**MPI clusters at T1 (m,s)**		
	**ID n = 49**	**AC n = 51**	**DYS n = 104**	**ID n = 55**	**AC n = 66**	**DYS n = 83**
Pain severity (100 = most)	75.9 (15.1)	63.0 (17.0)	82.6 (12.7)	76.4 (12.1)	59.5 (15.2)	83.6 (9.4)
Interference (100 = worst)	77.2 (13.0)	58.9 (16.4)	81.3 (10.9)	76.6 (11.7)	57.5 (15.2)	81.7 (9.7)
Life control (100 = best)	42.2 (17.6)	65.2 18.2)	45.1 (22.2)	42.7 (19.2)	63.7 (17.0)	44.4 (20.9)
Negative mood (100 = worst)	66.3 (13.6)	39.8 (17.3)	68.9 (15.0)	67.3 (14.9)	38.0 (17.3)	68.8 (18.0)
Support (100 = most)	44.0 (23.5)	61.9 (19.7)	85.0 (13.7)	41.6 (24.0)	66.9 (18.5)	88.7 (12.0)
Negative, punishing responses (100 = most)	34.4 (29.1)	11.0 (13.8)	28.6 (26.5)	33.0 (29.2)	13.9 (15.3)	26.9 (24.1)
Solicitous responses (100 = best)	33.3 (17.1)	47.5 (20.0)	75.5 (15.2)	34.5 (16.7)	53.0 (19.0)	79.0 (15.5)
Distracting responses (100 = best)	30.5 (18.7)	43.0 (21.2)	69.7 (17.6)	33.7 (20.5)	50.5 (19.4)	73.5 (16.9)
General activity (100 = most)	34.1 (12.0)	38.1 (12.1)	33.0 (14.9)	37.5 (11.6)	38.2 (12.5)	30.9 (14.3)

MPI: Multidimensional Pain Inventory, ID: MPI cluster interpersonally distressed, AC: MPI cluster adaptive copers, DYS: MPI cluster dysfunctional, T0: 4 weeks before entry to pain management program, T1: at entry to pain management program, m: mean, s: standard deviation.

**Figure 1:**
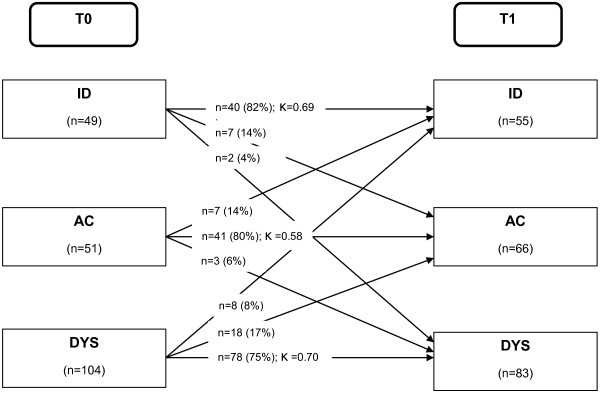
Multidimensional Pain Inventory subgroup classification changes T0: 4 weeks before entry to pain management program, T1: at entry to pain management program, MPI: Multidimensional Pain Inventory, ID: MPI cluster interpersonally distressed, AC: MPI cluster adaptive copers, DYS: MPI cluster dysfunctional, K: Cohen's Kappa coefficient.

At retest after 4 weeks, 82% of the patients in the Multidimensional Pain Inventory cluster interpersonally distressed (κ = 0.69), 80% of the adaptive copers (κ = 0.58), and 75% of the dysfunctional patients (κ = 0.70) did not change classification profile (Figure 1). Over the whole sample, 159 patients (78%) had a stable MPI subgroup classification. But, 22% of the patients (n = 45) did change Multidimensional Pain Inventory cluster group at retest. Most of the retest classification changes occurred in the subgroup adaptive copers: 18 dysfunctional patients (17%) and 7 interpersonally distressed patients (14%) were classified as adaptive copers at retest. Least retest classification changes took place in the dysfunctional subgroup (4% of interpersonally distressed patients and 6% of the adaptive copers).

## Discussion

In this study, we were able to provide additional evidence about the clinimetrical properties of the Multidimensional Pain Inventory. Testing it in 204 patients with chronic musculoskeletal pain – mainly chronic nonspecific back pain – demonstrated that test-retest reliability at scale level of the German version of the Multidimensional Pain Inventory was moderate to good and comparable to other language versions. The Multidimensional Pain Inventory Classification System – classifying patients into predefined subgroups labelled as adaptive copers, dysfunctional, and interpersonally distressed – is substantially stable.

### Reliability of scales compared to other versions

In a sample of 185 patients (60% chronic low back pain) Flor and colleagues measured an internal consistency score for the scale general activities of α = 0.63 [9]. In our sample we found for the same scale the superior score of α = 0.82. Test-retest reliability at an average 4-week time interval of the mean Multidimensional Pain Inventory scores at scale level yielded correlation coefficients between r_p_ = 0.73 and 0.87, making it on average better than the original German version and comparable with the original US version and several other language versions (Table 4). For this benchmark, the test-retest correlation coefficient for life control in our sample (r_p_ = 0.57) was lower.

**Table 4:** Comparison of different versions of the Multidimensional Pain Inventory concerning test-retest reliability of scales

**Pearson correlation coefficients (r**_**p**_**)**						
**MPI subscales**	**Original German version **[9]** (n = 25)**	**German cross-validation: current study (n = 204)**	**Original US version **[16]** (n = 60)**	**US cross-validation **[11]** (n = 199)**	**Swedish version **[26]** (n = 54)**	**Dutch version **[27]** (n = 59)**
Pain severity	0.59	0.77	0.82	0.74	0.75	0.74
Interference	0.78	0.82	0.86	0.87	0.85	0.89
Life control	0.72	0.57	0.68	0.62	0.81	0.74
Negative mood	0.68	0.73	0.69	0.53	0.74	0.73
Support	0.46	0.85	0.80	0.84	0.88	0.88
Negative responses	0.70	0.75	0.84	0.79	0.75	0.81
Solicitous responses	0.72	0.87	0.89	0.83	0.73	0.78
Distracting responses	0.89	0.77	0.62	0.80	0.76	0.65
General activity	0.73	0.86	0.89	0.85	0.80	0.78

### Interpretation of MPI classification changes

Most of the retest classification changes occurred in the subgroup adaptive copers: 17% of the dysfunctional patients and 14% of the interpersonally distressed patients were at retest classified in the less disabled subgroup of adaptive copers (Figure 1). This change in the subjective pain experience of the patients in this sample occurred within a 4-week period, although no therapeutic interventions took place. We hypothesize that anticipation of participation in a pain management program might have a positive effect on the mental health of the patients (improvement of locus of control, reduction of anxiety and depression), and so explains why a substantial number of dysfunctional and interpersonally distressed patients change at retest into the more favorable adaptive copers cluster.

### Stability of MPI Classification System compared to other research samples

This study partly challenges the results of three other studies assessing the test-retest stability of the MPI Classification System [11-13]. In the two samples of fibromyalgia up to one third of the patients changed Multidimensional Pain Inventory classification at retest (Table 5). These authors concluded that for a sizeable number of chronic pain patients, Multidimensional Pain Inventory classifications may not be stable and need to be reconsidered [11,13]. Our data on test-retest stability in patients with predominantly chronic back pain suggest that the MPI Classification System is according the definition of the kappa values of 0.58-0.70 substantially stable: only 22% of patients with predominantly chronic back pain who completed the Multidimensional Pain Inventory and who had been classified into one of the empirically derived subgroups altered their responses sufficiently to be classified into a different pain coping style after a four week time interval without therapeutic interventions. Our results are in line with the score changes of 28% of the predominantly low back pain patients in the study by Junghaenel & Broderick [12]. Further studies are needed to replicate these results in other musculoskeletal pain disorders. So far, the German version of the Multidimensional Pain Inventory can be recommended for reliable and stable classification of subgroups of patients with chronic back pain in observational studies and randomized controlled trials.

**Table 5:** Comparison of this sample with prior research samples investigating Multidimensional Pain Inventory subgroup stability

**Authors**	**Number of patients**	**Main diagnosis**	**Average time between test-retest**	**Percentage of unstable patients at retest**
Current study	n = 204	Chronic back pain (82%)	28 days	22%
Junghaenel & Broderick [12]	n = 99	Low back pain (84%)	14 days	28%
McKillop & Nielsen [13]	n = 376	Fibromyalgia (100%)	56 days	33%
Broderick et al [11]	n = 199	Fibromyalgia (100%)	27 days	35%

### Comparison of MPI classification system with other back pain classification systems

The Treatment Based Classification developed by Delitto and colleagues and the O’Sullivan Classification System are validated physiotherapy movement-based classification approaches to low back pain [28,29]. In these classifications, analysis of mainly mechanical spinal loading strategies and modified spinal movement strategies determines subgrouping. In accordance with the MPI Classification System, both classifications also assess certain psychosocial aspects (fear-avoidance behavior, and/or maladaptive pain behavior). Comparison of percent agreement scores (75–82%) and kappa coefficients (0.58–0.70) for test-retest stability of the MPI Classification System reveals scores that are at least as good as the corresponding scores of the widely accepted Treatment Based Classification and the O’Sullivan Classification System (31–97% agreement, κ = 0.11–0.80) (Table 6).

**Table 6:** Chance-corrected measures of agreement of different back pain classification systems comprising psychosocial aspects

**Classification system**	**No. of clusters**	**Percent agreement**	**Kappa coefficient (κ)**
MPI Classification System	3	75–82%	0.58–0.70 (current study)
Treatment Based Classification	3	65%	0.49–0.56 [30]
		76%	0.60 [31]
		31–55%	0.14–0.45 [32]
		79–81%	0.14 [33]
		58–90%	0.11–0.77 [34]
O’Sullivan Classification System	5	70–97%	0.61 (0.47–0.80) [35]
		73–92%	0.65 (0.57–0.74) [36]

MPI: Multidimensional Pain Inventory.

### Strengths and limitations of this study

The present study has several strengths: a large sample size, no missing data, and the use of a valid assessment tool implemented worldwide. On the other hand, a limitation of the study was that the patient sample was somewhat heterogeneous with 82% chronic nonspecific back pain, 15% fibromyalgia and 3% other medical diagnoses. This is a possible source of variance that may complicate the analysis, but the reliability and stability analyses compared scores for the same patient, a procedure which is not affected by the heterogeneity of the sample.

## Conclusions

The reliability of the German Multidimensional Pain Inventory was moderate to good and comparable to other language versions. Multidimensional Pain Inventory subgroup classification is substantially stable in chronic back pain patients when compared to other diagnostic groups and other examiner-based subgroup classification systems. The MPI Classification System can be recommended for reliable and stable specification of subgroups in observational and interventional studies.

## Abbreviations

MPI: Multidimensional Pain Inventory; CA: Cronbach’s alpha reliability coefficient; 95%CI: 95% confidence interval; m: Mean scale score; s: Standard deviation; ICC: Intraclass correlation coefficient; n: Number of patients; T0: 4 weeks before entry to pain management program; T1: At entry to pain management program; ID: Multidimensional Pain Inventory cluster interpersonally distressed; AC: Multidimensional Pain Inventory cluster adaptive copers; DYS: Multidimensional Pain Inventory cluster dysfunctional.

## Competing interests

The authors declare that they have no competing interests.

## Authors’ contributions

MLV, FA, JBS, AA and RAB were responsible for the design of the study. AA procured funding. SL, RB and MLV collected the data. Statistical analysis was performed by FA and MLV. MLV and FA interpreted the data and made a first draft of the manuscript. All authors have read and approved the final manuscript.

## Pre-publication history

The pre-publication history for this paper can be accessed here:

http://www.biomedcentral.com/1471-2474/13/155/prepub
